# An Uncommon Cause of Hypertensive Urgency in Young Adolescent: Case Report

**DOI:** 10.5005/jp-journals-10071-23210

**Published:** 2019-07

**Authors:** Parag Shankarrao Dekate, Satyanarayana Reddy, VSV Prasad, Sudha Boda, Lokesh Saini, Prashant Patil

**Affiliations:** 1-6 Department of Pediatrics, PICU, Lotus Hospital for Women and Children, Lakdikapool, Hyderabad, Telangana, India

**Keywords:** Angiography, Anti-hypertensive, Mid aortic syndrome, Percutaneous dilatation

## Abstract

Mid aortic syndrome is rare cause of hypertensive urgency in children with poor outcome if left untreated, high index of suspicion with prompt management is the key to survival with good outcome. A 12-year-old boy was presented with fever, puffiness of face, and breathing difficulty. Clinically, he had hypertension with differential pulsation and BP in upper and lower limbs. He had peak systolic gradient of 80 mm Hg between upper and lower limb. His echocardiography and CT angiography was suggestive of significant isolated 80% narrowing of abdominal aorta without involvement any other large vessels. Percutaneous balloon dilatation of aorta was done considering multiple parameters. Post procedure, there was significant improvement in BP and we could wean his multiple anti-hypertensive drugs to keep his blood pressures below 95^th^ centile. His BP remained control with minimum upper and lower limb gradient on follow up of almost 1 year.

**How to cite this article:**

Dekate PS, Reddy S, Prasad VSV, Boda S, Saini L, Patil P. An Uncommon Cause of Hypertensive Urgency in Young Adolescent: Case Report. Indian J Crit Care Med 2019;23(7):339–341.

**Key message:**

Mid aortic syndrome is most uncommon amongst them. With prompt diagnosis and proper selection of therapeutic options like balloon dilatation or surgical correction, it has good prognosis. Aortic narrowing because of different diseases is an uncommon cause of HT urgency in children.

## INTRODUCTION

Narrowing of the abdominal aorta was first described by Quain in 1848,^1^ but the term middle-aortic syndrome (MAS) was originally used by Sen et al.^2^ It commonly involves renal (>80%) and splanchnic (50–70%) branches of the aorta.^3,4^ It is the commonest clinical syndrome associated with stenotic aorto-arteriopathy in children.^5^ Severe MAS is associated with significant morbidity and mortality. Almost 50% of untreated MAS patients develop hypertensive encephalopathy, congestive heart failure, and stroke in third or fourth decade, with less than 20% survival reported after age 40 years.^6–8^ Medical management of hypertension caused by MAS has been largely unsuccessful,^5,9^ and invasive intervention is often required to achieve adequate blood pressure control, relieve symptoms and to reverse or prevent end-organ damage.

We report a 12-year-old boy with an uncontrolled diagnosed as MAS and managed with balloon dilatation of abdominal aorta.

## CASE HISTORY

A 12-year-old boy presented with fever for 20 days, puffiness of face for 5 days, rapid breathing 3 days prior to admission.

At admission, he had features suggestive of congestive cardiac failure. He was treated decongestive measures like fluid restriction and diuresis. He had signs of failure to thrive with a weight of 18.9 kg (< 3^rd^ percentile) and a height of 125 cm (< 3^rd^ percentile). His blood pressure, measured several times, was in the range of 168/78 to 180/96 mm Hg which was more than 99^th^ centile; his pulse was around 129 beats/minutes regular sinus rhythm with all pulses well palpable. He had significant blood pressure gradient between upper and lower limb (Table 1).

On systemic examination, he had soft systolic murmur (grade 3/6) at precordium and hepatomegaly with liver span of 10 cm.

The child was started on intravenous antihypertensive drugs, while simultaneous attempts were made to identify the cause of this hypertension. The complete blood count, serum electrolytes, urea, creatinine, liver function tests and complete urine examinations were normal.

Later ECG, 2D Echo showed signs of left ventricular hypertrophy with LVEF of 40–45%. USG abdomen with renal Doppler was normal. His thyroid profile, renin, aldosterone levels and urine for catecholamines were normal.

**Fig. 1:**
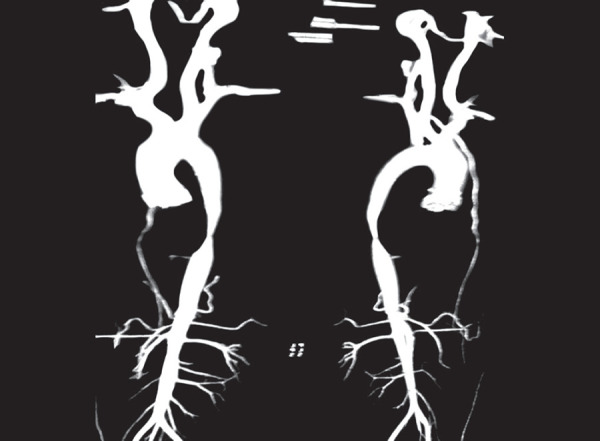
CT angiography of aorta and its branches along with CT abdomen

Based on differential blood pressures in upper limb and lower limb, we considered the possibility of some obstructive component in the Aorta, hence CT angiography of Aorta and its branches along with CT abdomen were done which showed 80% stenosis of abdominal aorta proximal to the origin of renal arteries without any stenosis of renal and mesenteric vessels (Fig. 1).

We investigated further to find out the acquired causes of MAS like neurofibromatosis, vasculitis disorders like Takayasu arteritis, retroperitoneal fibrosis or abdominal mass compressing aorta by performing genetic analysis, ANA, anti DsDNA, CRP, ESR, CT abdomen which were normal. Tuberculosis was also ruled out by real time DNA PCR, hence diagnosis of congenital MAS was considered in our patient.

**Table 1:** Table showing systolic, diastolic and mean blood pressure and different gradient between them (All BP in mm Hg)

*Location*	*SBP*	*DBP*	*Mean blood pressure*	*Mean blood pressure gradient*	*SBP* *gradient*
Right upper limb	164	60	94		
Left upper limb	162	65	96		
Left lower Limb	84	50	61	33	80
Right upper Limb	78	48	58	36	86

**Fig. 2:**
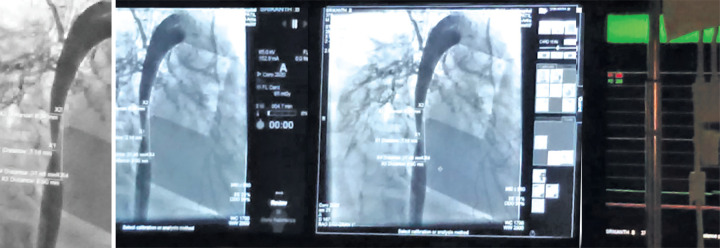
Aortic stenosis post-balloon dilatation

Percutaneous balloon dilatation was done in view of the growing age of child and isolated aortic involvement without involvement of renal/mesenteric vessels. The procedure was uneventful without any complications of vascular leaks, intimal tears (Fig. 2).

Postoperatively his lower limb blood pressures improved. We were able to titrate his anti hypertensive drugs, and he was discharged on oral antihypertensive drugs and followed up regularly.

On follow-up since last 1 year, his blood pressures were within normal range. Repeat CT angigraphy did not show any aneuryms or restenosis, but parents were counselled for definitive surgery (aorto-aortic bypass) at a later stage i.e., after completing 18–20 years of age.

## DISCUSSION

Most common location of coarcation of aorta is in the thoracic aorta either distal or proximal to ligamentum arteriosum. Middle aortic syndrome (MAS) is a rare entity characterized by localized narrowing of abdominal or distal thoracic aorta.^10^ It constitutes about 0.5–2% of all the cases of aortic coarctation.^11^

MAS may be congenital or acquired. Congenital coarctation has been thought to be due to incomplete fusion or overfusion of embryonic dorsal aortas during 4th week of gestation.^11–13^ Another hypothesis may be intra-uterine injury or infection, particularly rubella as the risk factor that precipitates aortic hypoplasia.^4,10^ Causes of acquired MAS are neurofibromatosis, William's syndrome, Alagille syndrome, fibromuscular dysplasia, retroperitoneal fibrosis (Ormond disease), mucopolysaccharidosis, foetal alcohol syndrome and giant cell arteritides including temporal (cranial) and Takayasu arteritis.^4,10^

MAS usually presents with hypertension in young age group refractory to medical therapy. Rarely, it presents as lower limb claudication or abdominal angina.^10^ The life expectancy of patients with untreated MAS is 30–40 years. The main reason of death is cardiovascular complications of progressive hypertension including cerebrovascular accidents, cardiomegaly, left heart failure and coronary artery disease.^10,14^

The severity of hypertension is the primary indicator for intervention and the factor determining procedural timing. Endovascular therapy may provide a sound minimally invasive treatment in MAS caused by discrete aortic stenosis that does not encompass the mesenteric and renal arteries.^10^ Balloon angioplasty and stenting as a palliative option to avoid surgery on the developing aorta has been described.^15–20^ Limitations of angioplasty and stenting include technical failures,^15,16^ iatrogenic tears and dissections,^16–18,20^ aneurysms^20^ and restenosis.^16^

Definitive surgery is the primary treatment of tubular aortic narrowing associated with renovascular hypertension and visceral artery stenosis. For patients with active vasculitis surgery is not recommended in active phase of disease.^10,14^ For children, best results are achieved if definitive treatment can be delayed till they have achieved full growth.

In our case, initially medical management failed. Percutaneous balloon dilatation was done in view of the growing age of child and isolated aortic involvement without involvement of renal/mesenteric vessels. The procedure was uneventful without any complications of vascular leaks, intimal tears. After 1 year of follow-up, his blood pressures were within normal range. Repeat CT angigraphy did not show any aneuryms or restenosis, but parents were counselled for definitive surgery (aorto-aortic bypass) at a later stage i.e., after completing 18–20 years of age.

## CONCLUSION

MAS is a rare cause of uncontrolled hypertension with poor outcome if left untreated. Balloon dilatation is effective in isolated aortic involvement but surgery is the definitive treatment at a later stage.
